# PETAL LOSS, a trihelix transcription factor that represses growth in *Arabidopsis thaliana*, binds the energy-sensing SnRK1 kinase AKIN10

**DOI:** 10.1093/jxb/erv032

**Published:** 2015-02-19

**Authors:** Martin O’Brien, Ruth N. Kaplan-Levy, Tezz Quon, Pia G. Sappl, David R. Smyth

**Affiliations:** School of Biological Sciences, Monash University, Melbourne, Vic. 3800, Australia

**Keywords:** AKIN10, *Arabidopsis*, Golgi, PETAL LOSS, SnRK1, transcription factor, trihelix.

## Abstract

Organogenesis in plants involves differential growth. Rapidly growing primordia are distinguished from the meristem and each other by slower growing boundaries. PETAL LOSS (PTL) is a trihelix transcription factor of *Arabidopsis* that represses growth in boundaries between newly arising sepals. To identify partners involved in this growth limitation, a young inflorescence cDNA library was screened by yeast two-hybrid technology with PTL as bait. The most frequent prey identified was AKIN10, the catalytic α-subunit of the Snf1-related kinase1 (SnRK1). Interaction was mapped to the C-terminal (non-kinase) half of AKIN10 and the N-terminal portion of PTL. Binding of PTL was specific to AKIN10 as there was little binding to the related AKIN11. The interaction was confirmed by co-immunoprecipitation *in vitro*. Fluorescently tagged products of 35S:YFP-AKIN10 and 35S:CFP–PTL also interacted when transiently expressed together in leaf cells of *Nicotiana benthamiana*. In this case, most of the cytoplasmic AKIN10 was preferentially moved to the nucleus where PTL accumulated, possibly because a nuclear export sequence in AKIN10 was now masked. During these experiments, we observed that AKIN10 could variably accumulate in the Golgi, shown by its co-localization with a tagged Golgi marker and through its dispersal by brefeldin A. Tests of phosphorylation of PTL by AKIN10 gave negative results. The functional significance of the PTL–AKIN10 interaction remains open, although a testable hypothesis is that AKIN10 senses lower energy levels in inter-sepal zones and, in association with PTL, promotes reduced cell division.

## Highlight

PETAL LOSS binds AKIN10 in yeast, *in vitro*, and in the nucleus when transiently co-expressed in leaves. Together they may act to reduce cell division in boundaries between developing sepals.

## Introduction

Organogenesis in plants is associated with the lateral outgrowth of primordia from the periphery of meristems. In the shoot apical meristem, boundaries develop between leaf primordia and the meristem as the leaves arise. In the flower meristem, similar boundaries occur between newly arising whorls of floral organs and the meristem, as well as between organs of the same type within whorls. A common property of boundary regions is a reduced rate of cell proliferation (Breuil-Broyer *et al.*, 2004). This is associated with reduced levels of the growth-promoting hormones auxin (Heisler *et al.*, 2005) and brassinosteroids (Gendron *et al.*, 2012).

The genetic control of boundary formation has been investigated. In most cases, the genes identified encode transcription factors (reviewed by Žádníková and Simon, 2014). One class encodes NAC transcription factors, involving three *CUP-SHAPED COTYLEDON* (*CUC1/2/3*) genes in *Arabidopsis* (Aida *et al.*, 1997). The expression of *CUC1* and *CUC2* is downregulated by auxin (Furutani *et al.*, 2004; Heisler *et al.*, 2005) and brassinosteroids (Gendron *et al.*, 2012). This does not occur in boundary regions where hormone levels are lower, thus allowing their probable function to repress cell division.

*PETAL LOSS* (*PTL*) is a boundary gene of *Arabidopsis* involved in promoting boundaries between developing sepals (Griffith *et al.*, 1999). It encodes a transcription factor of the trihelix family, and is most strongly expressed between newly arising sepals (Brewer *et al.*, 2004). Loss of PTL function results in some outward overgrowth of the inter-sepal zone (Lampugnani *et al.*, 2012). On the other hand, ectopic expression of PTL leads to suppression of growth in any tissue where it is abnormally present (Brewer *et al.*, 2004). Thus, PTL apparently normally limits cell division in the inter-sepal zone, acting to maintain an appropriate boundary. Genetic studies of interactions between PTL and CUC1 indicated that CUC1 functions differently, repressing upward rather than outward growth in the inter-sepal zone (Lampugnani *et al.*, 2012). Similar genetic investigation of PTL interactions with RABBIT EARS (RBE), a zinc-finger transcription factor active in perianth development, suggested that it also represses tissue growth in this region, although acting independently of PTL (Lampugnani *et al.*, 2013).

Despite its name, it seems likely that PTL influences petal initiation only indirectly. Some petals are initiated in early-formed flowers in *ptl* null mutant plants indicating that PTL function is not absolutely required (Griffith *et al.*, 1999). Also, *PTL* is not expressed in petal anlagen or primordia (Lampugnani *et al.*, 2012). Genetic interactions with auxin transport mutants revealed that loss of PTL function sensitizes the flower primordium to distortions in auxin dynamics such that almost no petals are formed in double mutants (Lampugnani *et al.*, 2013). Significantly, generation of auxin in the PTL expression zone can rescue petal initiation in *ptl* mutant plants. It may be that overgrowth of the inter-sepal zone in *ptl* mutants is responsible for secondarily disrupting the movement of auxin as a petal initiation signal.

During a search for partners of the PTL protein in defining the inter-sepal zone, we uncovered a specific protein–protein interaction with the energy-sensing kinase AKIN10. AKIN10 acts as a global sensor of energy deprivation, downregulating energy-requiring metabolism and triggering the activation of catabolic processes (Baena-González *et al.*, 2007). *AKIN10* encodes a kinase α-subunit of Snf1-related kinase1 (SnRK1), and is also known as *KIN10*, *SnRK1.1* and *SnRK1α1*. Snf1 itself is a sugar-non-fermenting protein kinase of yeast that plays a parallel role in regulating metabolism. The process is highly conserved, as equivalent AMP-activated kinases (AMPK1/2) also occur in mammals and are closely related in structure and function to Snf1 and SnRK1 (Polge and Thomas, 2007; Hedbacker and Carlson 2008; Halford and Hey, 2009; Ghillebert *et al.*, 2011; Hardie, 2012; Crozet *et al.*, 2014).

*AKIN10*, and another SnRK1 kinase α-subunit gene *AKIN11*, are expressed ubiquitously and at similar levels in *Arabidopsis*, although a third α gene, *AKIN12*, is expressed at lower levels and only in pollen, developing embryos, and seeds (Schmid *et al.*, 2005). AKIN10 is activated by specific upstream kinases in response to energy deprivation (Sugden *et al.*, 1999). This activation is inhibited by trehalose-6-phosphate, a signal of energy sufficiency (Zhang *et al.*, 2009). AKIN10 can then phosphorylate key metabolic enzymes to immediately influence their activity. In addition, it has a longer-term effect on global gene expression through its regulation of specific transcription factors, including several basic leucine zippers that bind G boxes in promoter regions of upregulated target genes (Baena-González *et al.*, 2007). The mechanism of activation of these bZIPs by AKIN10 is not clear, but AKIN10 has been shown to phosphorylate and activate FUSCA3 (FUS3), a transcription factor of the B3 class involved in embryo maturation (Tsai and Gazzarrini, 2012).

In the present study, AKIN10 and PTL were shown to bind each other in yeast, *in vitro*, and following transient expression *in planta*. In the latter, much of the cytoplasmic AKIN10 was relocalized to the nucleus, presumably bound to PTL, which accumulates there. The functional significance of this interaction was explored.

## Materials and methods

### Preparation of cDNA library

Wild-type Columbia (Col-0) plants were grown at 20 °C under short days (9h light/ 15h dark) for 30 d, and then transferred to long days (16h light/ 8h dark) for 14 d. Primary inflorescence tips with buds no older than stage 5 (Smyth *et al.*, 1990) were dissected from surrounding leaves and the basal stem using a 26-gauge needle and snap frozen in liquid N_2_. Total RNA was extracted using an RNeasy Plant Minikit (Qiagen) and treated with DNase I (Ambion). A cDNA library was generated using a Clontech Matchmaker Library Construction and Screening kit. Activation domain (AD)-tagged cDNA inserts in plasmid pGADT7-Rec were generated (~2.2×10^7^ clones) and amplified in yeast strain AH109.

### Yeast two-hybrid experiments

The Clontech Matchmaker Two-Hybrid System 3 was used. For library screening, binding domain (BD)-PTLΔC1 bait (plasmid pGBKT7) with the C-terminal AD removed (Kaplan-Levy *et al.*, 2014) in cells of yeast strain Y187 were mated with the AD-library prey (plasmid pGADT7) in strain AH109, and approximately 4×10^7^–1×10^8^ diploids selected for each mating. 3-Amino-1,2,4-triazole (3-AT; Sigma) was included to suppress leaky growth of His3p and any activation by bait sequences, with the required concentration determined by trial runs. A concentration of 2.5mM 3-AT was found to be sufficient to inhibit auto-activation by full- length BD–PTL. For pairwise interaction tests, a full-length clone of the protein-coding sequence of AKIN10 (At3g01090.2) was obtained from the Arabidopsis Biological Resource Center (U24028; see Supplementary Table S1 at *JXB* online for primer sequences). Full-length AKIN11 coding sequence (At3g29160) was obtained from the inflorescence cDNA library by reverse transcription (RT)-PCR and 5′-rapid amplification of cDNA ends. Truncations and deletions of PTL used to locate sites of interaction have been described elsewhere (Kaplan-Levy *et al.*, 2014). Activation was quantified using triplicate technical assays of α-galactosidase activity of the *MEL1* reporter in liquid cultures.

### Co-immunoprecipitation

Full-length PTL protein tagged with the c-Myc epitope and AKIN10 protein tagged with haemagglutinin were generated from inserts in pGBKT7 and pGADT7 plasmid clones, respectively (made for yeast two-hybrid screening), using rabbit reticulocytes (TnT T7 coupled system, Promega). AKIN10 was labelled with ^35^S-methionine (ICN) during synthesis. ^35^S-AKIN10 was incubated without and with Myc–PTL, and PTL was immunoprecipitated using Myc antibody following the instructions for the Matchmaker Co-IP kit (Clontech).

### Generation and expression of pAKIN10:GUS reporter lines

The 1035bp region from the stop codon of the upstream gene (At3g01100.1) to the first methionine codon in the second exon of AKIN10 (as in splice form At3g01090.2) was amplified from genomic DNA (see Supplementary Table S1 for primer sequences) and translationally fused with the first methionine of the β-glucuronidase (GUS) gene in plasmid pRITA. This full-length insert was labelled pAKIN10a:GUS. Three smaller versions of the insert were generated, carrying sequences from the 5′ end to the transcription start site of AKIN10 (526bp, pAKIN10b:GUS), from the end of the 3′-untranslated region of the upstream gene to the transcription start site (232bp, pAKIN10c:GUS), or from this start site to the 3′ end (509bp, pAKIN10d:GUS). The inserts were excised with *Not*I and inserted into pMLBART, transferred to *Agrobacterium tumefaciens* strain AGL1, and transformed into Columbia wild-type plants. For each insert, 12 independent T1 transformants were selected that yielded consistent GUS staining levels and patterns in at least seven transformants using a method reported previously (Brewer *et al.*, 2004). To avoid product leaching, 6mM potassium ferri- and ferrocyanide was included.

### Generation and transient expression of fluorescently tagged versions of AKIN10 and PTL

To test the interaction between AKIN10 and PTL proteins, our modification of intracellular localization (MILo) method was used (Kaplan-Levy *et al.*, 2014). This involves tagging potential partner proteins that occupy different cellular compartments with different fluorochromes. If the fluorescent tag of one protein is moved from one cellular compartment into that occupied by the other protein, we conclude that the two proteins bind stably.

The 35S:YFP–AKIN10 construct was made by amplifying the AKIN10 coding sequences in clone U24028 (see above) to generate an *Eco*RI site adjacent to the ATG start codon and a *Bam*HI site immediately downstream of the stop codon. This was inserted in frame downstream of the 35S–YFP sequence from plasmid pEYFP-C1 (Clontech) previously inserted in pART7 (Gleave 1992). The full reporter sequence was excised with *Not*I and inserted into pMLBART for transformation into *A. tumefaciens* strain AGL1. The 35S:CFP–PTL construct, and the 35S:CFPN7–PTL derivative generated from it, were made using the plasmids mCerulean-C1 and pART7 as described previously (Kaplan-Levy *et al.*, 2014).

Joint transfection of leaves of *Nicotiana benthamiana* with yellow fluorescent protein (YFP) and cyan fluorescent protein (CFP) plasmids in AGL1 followed the method of Kaplan-Levy *et al.* (2014). All experiments were carried out using duplicate leaves for each treatment, and were replicated on at least three occasions. Fluorescence imaging using a Zeiss Axioskop 2 MOT compound microscope was as reported elsewhere (Kaplan-Levy *et al.*, 2014).

### Intracellular location of AKIN10 following transient expression

The 35S:YFP–AKIN10 construct above was jointly transfected into leaves of *N. benthamiana* with the CFP-tagged Golgi marker plasmid G-ck obtained from the Arabidopsis Biological Resource Center. The latter carries the cytoplasmic tail and transmembrane domain of GmMan1, a soybean α-1,2-mannosidase (Nelson *et al.*, 2007), and was electroporated into *A. tumefaciens* AGL1 by Edwin Lampugnani (strain ERL292). Excised leaf segments were immersed either in distilled water alone or in water containing 50 μg ml^–1^ of brefeldin A (Genesearch). The latter (and water controls) were allowed to stand for 25–30min before viewing. Confocal images were collected using a Leica SP5 microscope. YFP images were obtained from fluorescence between 465 and 505nm excited using a 458nm laser line, and CFP images between 525 and 600nm using a 514nm laser line (both lines attenuated to 33%). Other details followed the method of Lampugnani *et al.* (2013).

### Kinase assay

PTL protein tagged with the c-Myc epitope was generated from the pGBKT7 plasmid clone using a rabbit reticulocyte system as above. The AKIN10 sequence was inserted into plasmid pQE30 (see Supplementary Table S1 for primer sequences), expressed in *Escherichia coli* strain M15 cells, and tagged with a 6×His epitope purified using nickel beads (Ni-NTA kit; Qiagen). The two protein preparations were incubated together with [γ-^32^P]ATP (ICN) and separated by 10% SDS-PAGE. Prior to Western blot transfer, the gel was wrapped in Clingwrap and autoradiographed. The gel was then blotted and the membrane treated with mouse monoclonal anti-Myc antibody (Co-IP kit; Clontech), followed by rabbit anti-mouse antibody tagged with horseradish peroxidase, which was detected by luminol chemiluminescence using an ECL Prime Western Detection kit (Amersham). A duplicate gel run in parallel was similarly blotted, treated with mouse monoclonal anti-His (Qiagen), and visualized as above.

## Results

### PTL binds AKIN10 in yeast and *in vitro*

**Fig. 1.**
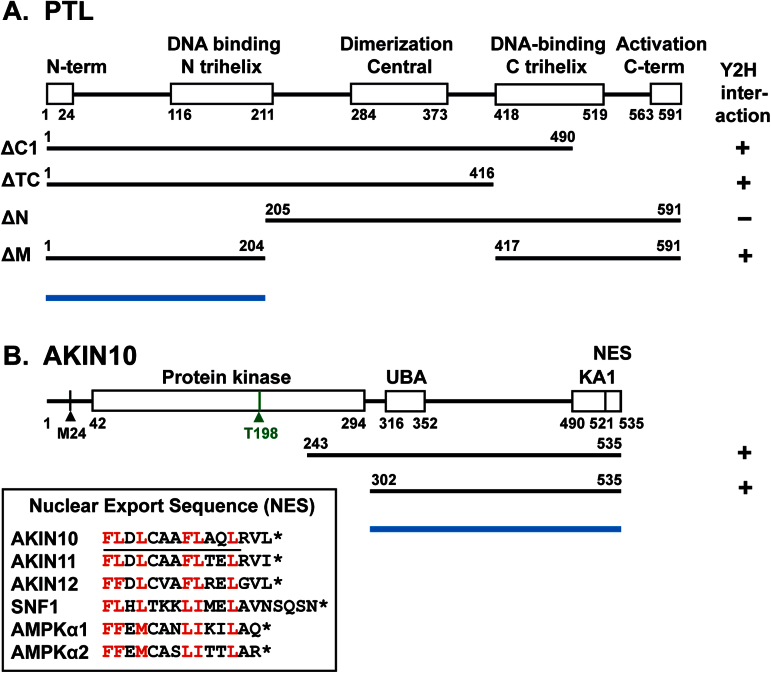
Maps of PTL and AKIN10, showing the results of yeast two-hybrid interactions. (A) Map of the PTL protein, showing the conserved trihelix DNA-binding domains, the central dimerization domain, and two terminal domains. BD–PTLΔC1, used in the library screen, removes a C-terminal activation region. The extent of other deletions used in yeast two-hybrid tests, and the results of these tests with AD–AKIN10, are shown. (B) Map of the AKIN10 protein, with functional domains predicted by Pfam. These are a kinase domain that includes a phosphorylated threonine at codon 198 (green), a ubiquitin-associated domain (UBA), and a C-terminal kinase-associated domain (KA1). The last 15 residues closely match a conserved nuclear export sequence (NES) (Kazgan *et al.*, 2010). Its sequence in *Arabidopsis* (AKIN10/11/12), yeast (SNF1), and human (AMPKα1/2) proteins is shown, with conserved bulky hydrophobic amino acids (L, I, F, V, and M) indicated in red, and the predicted amphipathic α-helical region underlined in AKIN10. The overall map is based on splice form At3g01090.2, which includes an additional 5′ exon. The first methionine of the product of two other splice variants, At3g01090.1 and At3g01090.3, is indicated (M24). The extent of two truncated cDNA clones from a cDNA library that interacted with PTL in yeast cells is shown. Blue bars indicate the regions of AKIN10 and PTL required for their interaction.

To search for partners of the PTL protein, yeast two-hybrid screens were performed. A cDNA library was made from dissected inflorescences with buds no older than stage 5 (Smyth *et al.*, 1990) and combined with the yeast GAL4 AD. This was co-expressed with BD–PTL∆C1 as bait, in which the endogenous PTL activation region at the C terminus had been deleted (Fig. 1A). An initial screen was carried out under stringent selective conditions for HIS3 activity (25mM 3-AT). Prey inserts of 12 strongly growing colonies were sequenced, and nine contained sequences encoding AKIN10 (At3g01090) (Fig. 1B). Three other screenings of the same library using the same bait under more relaxed selection conditions (5, 1, and 0mM 3-AT), and choosing colonies of a range of sizes, also yielded predominantly AKIN10 clones. Of the inserts in 48 colonies sequenced, eight were AKIN10, representing two different cDNA insertions, commencing at codon E243 or L302, the latter downstream of the kinase domain (Fig. 1B). When inserts of a larger sample of 447 clones from these three screenings were probed with an AKIN10-specific sequence, 97 (22%) hybridized.

**Fig. 2.**
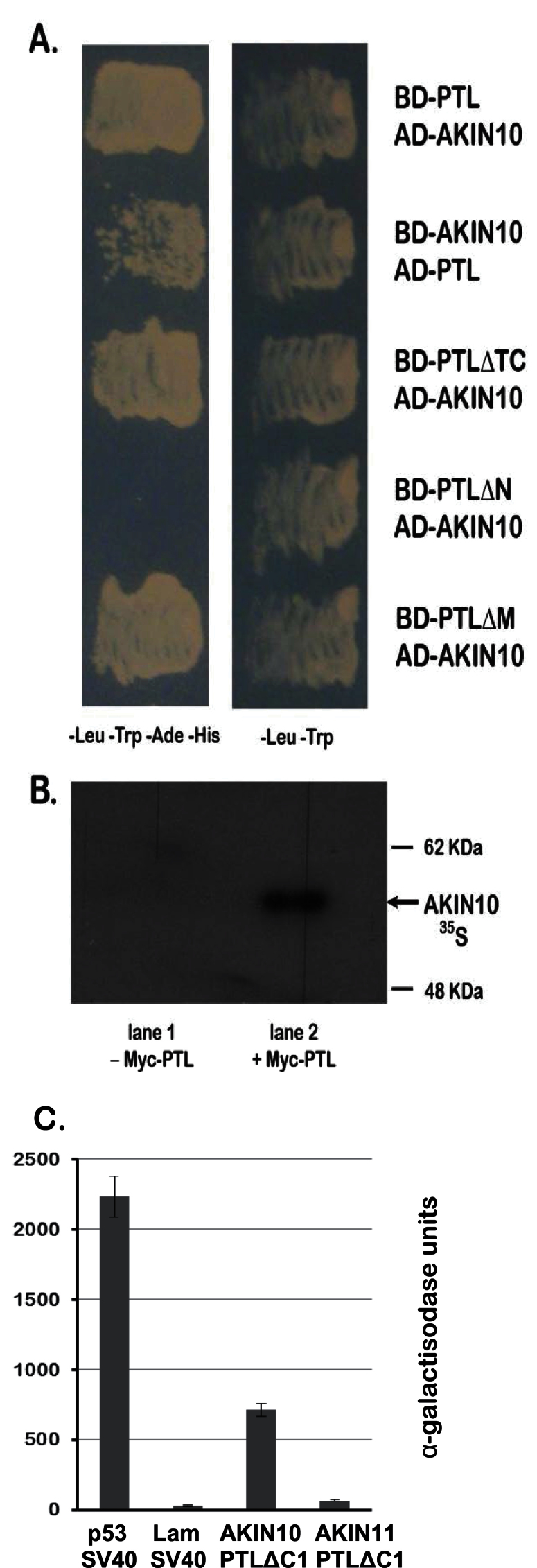
PTL interacts with AKIN10 in yeast and *in vitro*. (A) Yeast two-hybrid interactions of full-length and deleted versions of PTL (see Fig. 1) with AKIN10, showing that interaction does not occur if the N terminal third of PTL is deleted. Medium contained 25mM 3-AT to require strong histidine prototrophy for growth, and to inhibit autoactivation conferred by the C-terminal region of PTL (for negative controls, see Kaplan-Levy *et al.*, 2014). Left column: growth indicates interaction. Right column: control. (B) Autoradiograph showing co-immunoprecipitation of ^35^S-labelled AKIN10 with Myc-tagged PTL following treatment with anti-Myc antibody. The control (lane 1) lacks Myc–PTL. (C) Quantitative assay of yeast two-hybrid interaction between BD–PTLΔC1 and AD–AKIN11 showing a very weak interaction compared with AD–AKIN10. A positive control (p53 and T-antigen of simian virus 40) and a negative control (Lam and T-antigen) are also shown. Means of three assays±standard errors are plotted for each combination.

To further test the PTL–AKIN10 interaction, prey containing full-length coding sequences of AKIN10 (from splice variant 2) was generated (AD–AKIN10) and tested for interaction with full-length BD–PTL as bait. As this bait retained the PTL AD, we used 25mM 3-AT to prevent growth of control strains carrying the bait alone. BD–PTL and AD–AKIN10 showed strong growth, confirming their interaction (Fig. 2A). The reverse combination, with BD–AKIN10 as bait and full-length AD–PTL as prey, also activated reporter genes, although somewhat more weakly (Fig. 2A).

To locate the region within PTL that interacts with AKIN10 in yeast, several partially deleted versions (Fig. 1A) were tested as bait against AD–AKIN10 prey. Whereas deletions of the C-terminal region from codon 417 (ΔTC), or the mid-region (codons 205–416, ΔM), still showed a strong interaction, loss of the N-terminal region (up to codon 205, ΔN) did not (Fig. 2A). Thus, in yeast cells the interaction domains fall in the N-terminal third of PTL (Fig. 1A).

PTL and AKIN10 also interacted *in vitro*. This was established by co-immunoprecipitation of ^35^S-labelled AKIN10 with epitope-tagged Myc–PTL when challenged with an anti-Myc antibody (Fig. 2B).

### PTL interacts specifically with AKIN10

AKIN11 (At3g29160) encodes an alternative α-subunit of SnRK1. Its product is closely related to AKIN10, showing 82% identity and 90% similarity at the protein level (excluding exon 1 present in At3g01090.2). Both genes are ubiquitously expressed and at similar levels (Schmid *et al.*, 2005). However, AKIN11 was not detected in our screens, although we confirmed the presence of AKIN11 transcripts in the inflorescence cDNA pool by RT-PCR. Thus, we were interested to examine whether AKIN11 was also able to interact with PTL.

A full-length open reading frame of AKIN11 was cloned into the prey vector (AD–AKIN11) and combined with PTL bait lacking the activation region (BD–PTL∆C1). Growth of yeast cells carrying both constructs was assessed on selective medium through the activity of the α-galactosidase protein in the host yeast strain Y187 (Fig. 2C). AD–AKIN11 activation was more than 10 times lower than for AD–AKIN10, and only slightly higher than the negative control. Thus, a strong interaction with PTL is specific to AKIN10.

### AKIN10 is expressed in developing tissues and overlaps with PTL expression domains in floral primordia

**Fig. 3.**
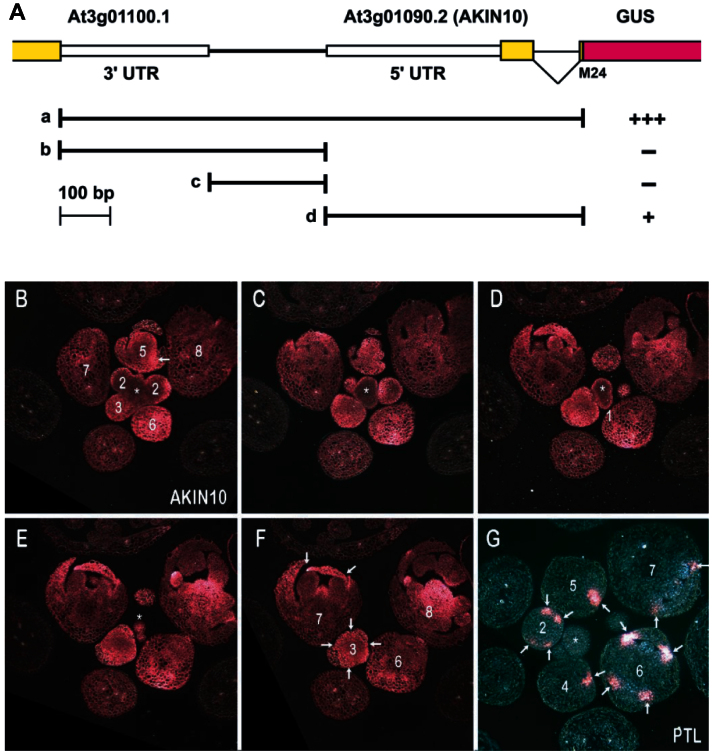
Expression patterns of AKIN10 and PTL in developing buds. (A) Design of the pAKIN10:GUS reporter constructs. Exons are shown in yellow and the GUS reporter in magenta. Solid lines indicate the four genomic regions used to drive GUS expression (a–d). Results summarized from 12 independent transformants of each are shown: +++, strong expression; +, weak expression; –, expression not detected (for examples,see Fig. S1). (B–F) Selections from transverse serial sections of an inflorescence expressing the full-length pAKIN10a:GUS. The product of the GUS reporter is blue but appears magenta under dark-field illumination. (G) Transverse section of an inflorescence expressing PTL (pPTL2.0i:GUS; see Brewer *et al.*, 2004). Asterisks indicate the inflorescence meristem, and numbers show the stages of flower development (Smyth *et al.*, 1990). Arrows in (G) indicate sites of PTL expression. These are also indicated in buds of equivalent stage from the AKIN10 expression series, or at earlier or later stages. [Note that sections of buds at stages 4 and 5 in (G) have intersected only one of the four PTL-expressing inter-sepal zones.]

For AKIN10 and PTL to physically interact, the transcripts and hence proteins must accumulate in the same cells (if they do not move between cells). To determine the tissue expression pattern of AKIN10 in developing buds, the upstream promoter region (including the 3′-untranslated region of the adjacent gene) and transcribed AKIN10 sequences down to the methionine in the second exon (codon 24) were translationally fused with GUS (Fig. 3A) and inserted into plants. Regions both upstream and downstream of the transcriptional start site were found to be required for strong expression (Fig. 3A and Supplementary Fig. S1 at *JXB* online).

AKIN10 was widely expressed, especially in newly developing tissues (Fig. 3B–F). It was only weakly expressed in the inflorescence meristem and bud primordia up to stage 2, except for the epidermis. Later, it was more strongly and evenly expressed throughout buds as floral organs developed (sepals from stage 3; petals and stamens from stage 5). As the organs grew out from stage 7, expression remained relatively high, although at lower levels in mature tissues.

By contrast, PTL expression in inflorescences was more restricted (Fig. 3G) (Brewer *et al.*, 2004). It was not expressed in the inflorescence meristem itself. In flower primordia, it was expressed in regions associated with reduced growth, at first on each side of the flower primordium from stage 1, and then internal to the anlagen of the lateral sepals at stage 2 (Fig. 3G, arrows). Its strongest expression occurred as four spots around the equator of the bud between newly arisen sepal primordia from stage 3 to stage 6. From stage 6 to stage 9, it was also expressed at the basal margins of developing sepals. All these regions corresponded with significant AKIN10 expression (Fig. 3B–F), although AKIN10 expression also occurred more widely.

### AKIN10 accumulates in the cytoplasm (including the Golgi) and variably in nuclei when transiently expressed in tobacco leaves

**Fig. 4.**
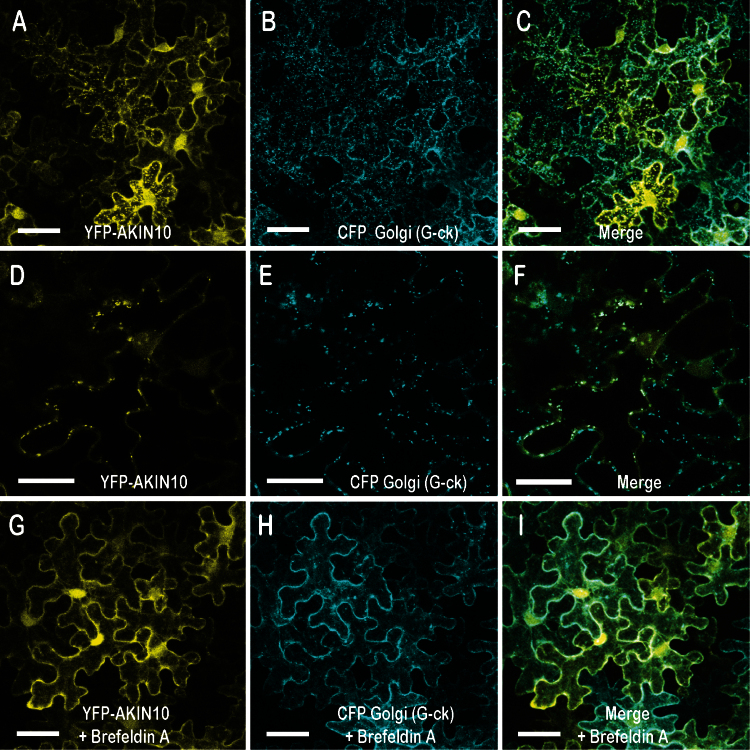
Intracellular localization of AKIN10 in the cytoplasm (including the Golgi) and in nuclei. 35S:YFP-AKIN10 and the Golgi marker 35S:CFP-G-ck were jointly transfected into leaves of N. *benthamiana*, and fluorescence was viewed by confocal microscopy. (A–C) Accumulation of AKIN10 (A) occurred in the plasma membrane region and the cytosol, and more variably in punctate bodies that coincided with Golgi (B, C). Some accumulation also occurred in nuclei. (D–F) Higher magnification images from a time course (for full series, see Supplementary Movie S1 at *JXB* online), showing the coincidence of AKIN10 and CFP-G-ck in one cell. (G–I) Images from the same experiment as (A–F) but with incubation of leaf segments in brefeldin A, a Golgi inhibitor, for 25–30min before imaging, showing loss of punctate bodies. Images in (A–C) and (G–I) are three-dimensional reconstructions of *z*-stacks, and in (D–F) are single optical sections. Bars, 20 μm (A–C, G–I); 10 μm (D–F).

Before testing if PTL and AKIN10 proteins associate when both are transiently expressed in plant tissues, the cellular localization of AKIN10 was examined, as this is the subject of conflicting reports (Fragoso *et al.*, 2009; Bitrián *et al.*, 2011; Tsai and Gazzarrini, 2012; Williams *et al.*, 2014). The fluorescently tagged construct 35S:YFP–AKIN10 was Agro-infiltrated into leaves of *N. benthamiana* and expression was observed by confocal microscopy. Significant accumulation was observed close to the plasma membrane, as well as more widely in the cytoplasm (Fig. 4A). Nuclear accumulation was also consistently seen. This was somewhat variable in relative intensity among experiments, although replicate leaves within an experiment gave closely similar results. Nuclear localization was not anticipated because AKIN10 carries a conserved nuclear export sequence (NES), as found in orthologous Snf1 and AMPK kinases from yeast and mammals (Fig. 1B) (Kazgan *et al.*, 2010).

In some cells, fluorescent punctate bodies were visible in the cytoplasm (Fig. 4A, D). On occasion, they were seen to move rapidly along linear pathways, and this behaviour, in addition to their number and size, suggested that they were Golgi bodies. To test this, we co-infiltrated 35S:YFP–AKIN10 with d35S:G-ck, a fluorescent CFP marker specific for Golgi apparatus (Fig. 4B, E) (Nelson *et al.*, 2007). Merged images showed that the punctate bodies coincided with the Golgi (Fig. 4C, F; Supplementary Movie S1). Furthermore, the bodies were lost if the excised infiltrated leaf segments were incubated in brefeldin A, a specific disruptor of Golgi structure (Satiat-Jeunemaitre and Hawes, 1992), before viewing (Fig. 4G–I). Thus, AKIN10 can accumulate in the Golgi when transiently expressed in leaf pavement cells.

### PTL and AKIN10 also interact when transiently co-expressed in tobacco leaves

35S:YFP–AKIN10 is found predominantly in the cytoplasm, whereas PTL accumulates in the nucleus (Kaplan-Levy *et al.*, 2014). To test if the two proteins bind each other, we examined their intracellular localization when co-expressed in the same cell to determine if they both now occupied the same compartment.

**Fig. 5.**
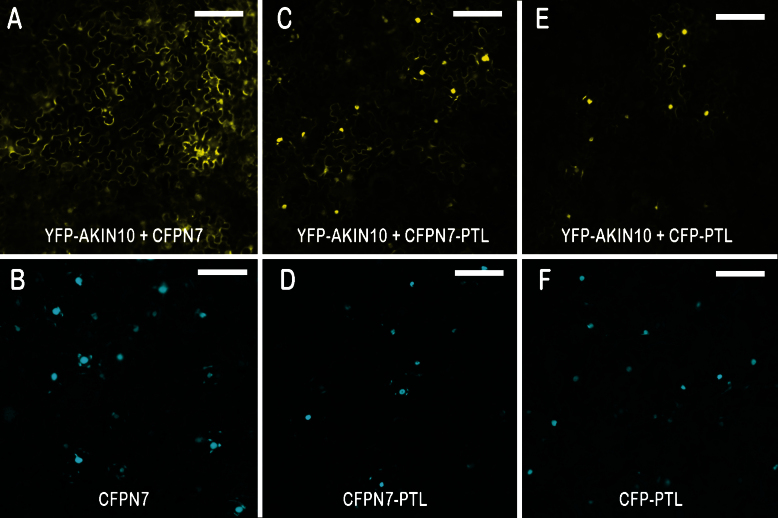
Interaction of AKIN10 and PTL following transient expression in tobacco leaves. Heterodimerization of AKIN10 and PTL was tested using the MILo method (Kaplan-Levy *et al.*, 2014). AKIN10 tagged with YFP was co-expressed with nuclear-localized CFP in leaves of *N. bethamiana*, and fluorescence was viewed by fluorescence microscopy. (A, B) As a negative control, 35S:YFP–AKIN10 was co-expressed with the nuclear-localized marker 35S:CFPN7 [shown when expressed alone in (B)]. YFP fluorescence remained predominantly cytoplasmic (A). (C, D) When 35S:YFP–AKIN10 was co-expressed with 35S:CFPN7–PTL (D), YFP was now mostly nuclear (C), indicating binding of AKIN10 and PTL and retention of the bound proteins in the nucleus. (E, F) When 35S:YFP–AKIN10 was co-expressed with 35S:CFP–PTL [lacking the supplementary NLS N7 but still nuclear (F)], YFP was still mostly nuclear (E), showing that PTL sequences were sufficient to co-localize AKIN10 in the nucleus. Bars, 100 μm.

To differentially tag PTL and to ensure its strong nuclear localization, we added CFPN7 (CFP carrying the strong nuclear localization sequence N7; Cutler *et al.* 2000) to generate 35S:CFPN7–PTL. First, we showed that 35S:CFPN7 alone (i.e. without the PTL sequence) (Fig. 5B) did not affect localization of the YFP fluorescence pattern associated with AKIN10 (Fig. 5A, compare with Fig. 4A). However, when PTL was added (35S:CFPN7–PTL) (Fig. 5D), the YFP–AKIN10 fluorescence was now much stronger in the nucleus (Fig. 5C). To model the natural situation more closely, we generated a 35S:CFP–PTL construct without the added N7 NLS, thus relying on the endogenous NLS sequences in PTL (Fig. 5F) (Kaplan-Levy *et al.*, 2014). In this case, too, the PTL sequences were able to localize much of the otherwise cytoplasmic AKIN10 to the nucleus (Fig. 5E). The conclusion is that AKIN10 and PTL proteins interact when both are transiently expressed in *N. benthamiana* leaves, and that the AKIN10 protein is predominantly carried into and retained in the nucleus as a consequence.

### Phosphorylation of PTL is not detected *in vitro*

**Fig. 6.**
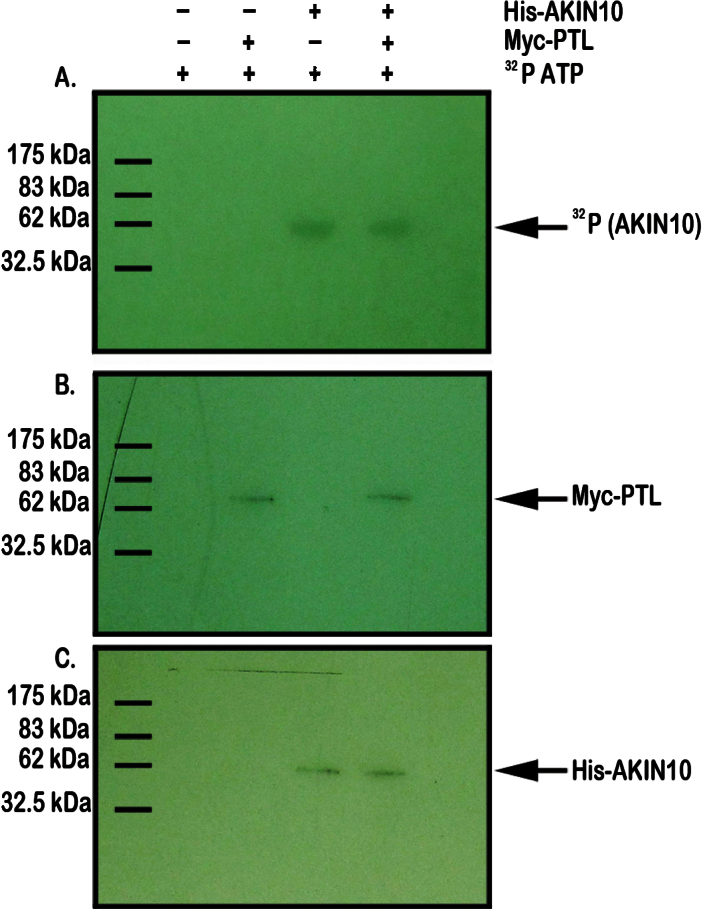
Test of phosphorylation of PTL by AKIN10. Mixed extracts of epitope-tagged AKIN10 and PTL proteins were incubated with [γ-^32^P]ATP and separated by gel electrophoresis. (A) Autoradiograph showing ^32^P-labelling of His–AKIN10-sized bands in lanes 3 and 4, but no labelling of Myc–PTL-sized bands in lanes 2 and 4, indicating that AKIN10 alone has been phosphorylated. (B) Western blot of the same extracts probed with anti-Myc antibody showing the presence of tagged PTL protein in lanes 2 and 4. (C) Western blot of the same extracts probed with anti-His antibody showing the presence of tagged AKIN10 protein in lanes 3 and 4.

As a kinase, AKIN10 may bind PTL in order to phosphorylate it. To test this, AKIN10 protein, tagged with a 6×His epitope tag, was produced in *E. coli* and purified. It was then combined with a preparation of PTL protein tagged with a c-Myc epitope made using a rabbit reticulocyte translation system, and the combination was supplied with [γ-^32^P]ATP. Phosphorylation of AKIN10 was required for its activation, and phosphorylation of a protein band the size of AKIN10 was clearly apparent in this system (Fig. 6A, C). However, there was no indication of label associated with any protein similar in size to epitope-tagged PTL (Fig. 6B).

## Discussion

### PTL and AKIN10 proteins specifically bind each other

We have shown that the PTL protein binds AKIN10 under three conditions: in yeast, *in vitro*, and when co-expressed in leaf pavement cells of *N. benthamiana.* The functional significance of this interaction was tested in several ways.

One possibility is that PTL is a substrate of the AKIN10 kinase. However, tests of phosphorylation of PTL by AKIN10 proved negative, although AKIN10 itself was probably phosphorylated in the *in vitro* test system used. PTL lacks the strict consensus recognition sequence of AMPK/SnRK1 serine/threonine kinases (Weekes *et al.*, 1993), and it may instead act as a bridge between AKIN10 and another component that is phosphorylated. A second transcription factor, FUSCA3, also binds AKIN10 in its C-terminal half (Tsai and Gazzarrini, 2012). In this case, phosphorylation of FUSCA3 by AKIN10 has been demonstrated, and a functional link was shown to be likely, as the growth consequences of overexpression of AKIN10 were reduced in plants that had lost FUSCA3 function. Similar tests of the effect of loss of PTL function in AKIN10 overexpressing plants would be worthwhile.

Functional significance was indicated by the finding that PTL bound AKIN10 but not AKIN11 in yeast two-hybrid tests. This specificity may reflect functional differences and depend on sequence divergence in the two α-kinase subunits. Both AKIN10 and AKIN11 carry strongly conserved ubiquitin-binding domains just downstream of the kinase domain (Farrás *et al.*, 2001), and kinase-associated domains (KA1) near the C terminus that interact with the β-subunits. They also share an NES at the C terminus (Fig. 1B; Kazgan *et al.*, 2010). However, there is a region of around 130 aa between the ubiquitin-binding domains and KA1 domains (Fig. 1B) that has lower sequence conservation (64% identical, 72% similar) (Bhalerao *et al.*, 1999) that may be responsible for the PTL binding specificity. Even so, in other yeast two-hybrid tests, AKIN10 and AKIN11 bind equally well to most members of the domain of unknown function DUF581 family of proteins that contain a putative C4 zinc finger (Nietzsche *et al.*, 2014). Furthermore, functional studies of AKIN10 and AKIN11 suggest that they act redundantly in energy sensing (Baena-González *et al.*, 2007), although consequences of their overexpression may differ somewhat (Williams *et al.*, 2014). Further comparison of AKIN10 and AKIN11 binding with PTL *in vitro* and in transient expression assays are needed. It would also be worthwhile testing the specificity of AKIN10 and AKIN11 binding to other trihelix proteins, related to PTL but differing in the N-terminal binding region (Kaplan-Levy *et al.*, 2012,^2014^).

If AKIN10 and PTL together regulate inter-sepal zone growth, then similar single mutant phenotypes would be expected. Loss-of-function phenotypes of AKIN10 have not been reported to date, and T-DNA insertion null mutants were not available at the time of this study (Tsai and Gazzarrini, 2012). Knockdown of AKIN10 function by RNA interference did not show an obvious abnormal phenotype (Baena-González *et al.*, 2007), although subtle petal defects may have been missed. Examination of *akin10* loss-function effects on petal initiation, and interactions with *ptl* loss-of-function mutants, are clearly a high priority for future work.

### PTL and AKIN10 each downregulate growth

Despite the lack of any firmly established functional significance, a link between the role of AKIN10 in sensing reduced energy levels with consequent downregulation of growth and the established role of PTL in repressing growth remains a realistic hypothesis.

PTL normally dampens cell division as loss of its function leads to a 35–40% increase of the inter-sepal zone (measured radially) during early stages of flower development without any increase in cell size (Lampugnani *et al.*, 2012). Conversely, ectopically expressed PTL blocks cell division, even when overexpressed in its usual locations (Brewer *et al.*, 2004). AKIN10, too, may act to dampen growth. Whereas downregulation of AKIN10 expression does not seem to affect morphogenesis, its upregulation results in delays to flowering and other phase transitions (Baena-González *et al.*, 2007), as well as growth defects in cotyledon and floral organ morphogenesis (Tsai and Gazzarrini, 2012). It is interesting that disruption of trehalose-6-phosphate levels, a signalling molecule acting negatively on SnRK1 and directly upstream of it (Zhang *et al.*, 2009), also disrupts site-specific growth. Enzymatic degradation of trehalose-6-phosphate is encoded by the *RAMOSA3* (*RA3*) gene in maize, and *ra3* mutants show increased axillary development within inflorescences (Satoh-Nagasawa *et al.*, 2006). *RA3* is specifically expressed nearby, and the additional T6P accumulating in *ra3* mutants might strongly inhibit SnRK1, thus allowing additional growth because determinacy of the axillary shoot is released.

To limit cell division, cyclins or cyclin-dependent kinases could be inactivated, or cell-cycle inhibitors activated. In this regard, the zinc-finger transcription factor JAGGED helps maintain growth of developing petals by repressing expression of two Kip-related cell-cycle inhibitors, KRP2 and KRP4 (Schiessl *et al.*, 2014), as well as also directly repressing *PTL* expression (Sauret-Güeto *et al.*, 2013). Global expression screens following AKIN10 activation have not identified cell-cycle regulatory targets, although mature leaf protoplasts were used that were not undergoing cell division (Baena-González *et al.*, 2007). Recently, AKIN10 was shown to phosphorylate and inactivate two other Kip-related proteins of *Arabidopsis*, KRP6 and KRP7 (Guérinier *et al.*, 2013). However, these proteins act as cyclin-dependent kinase inhibitors so their inactivation would be likely to result in continued cell division rather than its abolishment. The significance of such AKIN10–KRP interactions *in planta* needs further investigation.

### AKIN10 can accumulate in the Golgi as well as more widely in the cytoplasm and nuclei

In this study, we confirmed that AKIN10 is more strongly expressed in newly arising and growing organs than in mature differentiated tissues (Bitrián *et al.*, 2011; Williams *et al.*, 2014). Our inflorescence results also extend the observation made in tomato (Pien *et al.*, 2001) that a kinase subunit gene (*LeSNF1*) is not expressed significantly in the shoot meristem except for the epidermis.

The intracellular localization of AKIN10 has been variously reported. Bitrián *et al*. (2011) inserted a large genomic fragment including tagged AKIN10 coding sequence into transgenic *Arabidopsis* plants by recombineering. Although the results were reported at low optical resolution, fluorescence in most growing tissues seemed strongest in the region of the plasma membrane and in a cytoplasmic cloud surrounding the nucleus. Usually, it was not clearly apparent inside the nucleus itself. A similar lack of nuclear accumulation was reported in transgenic *Arabidopsis* plants expressing 35S:AKIN10 (Williams *et al.*, 2014). Our results, following transient expression in mature leaves of *N. benthamiana*, revealed accumulation at the plasma membrane and more widely in the cytoplasm, and some nuclear accumulation. Cytoplasmic and nuclear localization has also been reported in similar transient expression studies in intact leaves (Tsai and Gazzarrini, 2012; Williams *et al.*, 2014), leaf protoplasts (Cho *et al.*, 2012), and onion epidermal cells (López-Paz *et al.*, 2009). Thus, nuclear accumulation has been seen consistently in transient assays but less so in stable transgene experiments, and such differences may result from higher transient expression levels resulting in cytoplasmic saturation and consequent movement into the nucleus.

We have evidence that small fluorescent punctate bodies seen by us and others in 35S:AKIN10 transient expression experiments (López-Paz *et al.*, 2009; Tsai and Gazzarrini, 2012; Williams *et al.*, 2014) reflect Golgi bodies. We found that their occurrence is variable between cells and may reflect differing physiological conditions. Consistent with this, Williams *et al.* (2014) reported that similar bodies (‘small puncta’) developed rapidly in tissues damaged by cutting in both transient and stable expression assays. Tsai and Gazzarrini (2012) observed similar bodies but did not identify them, although they excluded plastids. This is in contrast to a report that 35S:AKIN10–GFP in transgenic *Arabidopsis* plants is associated with chloroplasts (Fragoso *et al.*, 2009), although different expression systems were involved. Williams *et al.* (2014) also excluded plastids, but they further excluded Golgi, peroxisomes, and mitochondria. Our finding of co-localization of punctate bodies with Golgi is inconsistent with this. It may be that rapid movement of the particles, apparent in time-course images (see Supplementary Movie S1), has masked their correspondence with a Golgi marker.

It is possible that AKIN10 trafficking in Golgi is simply an abnormal consequence of high levels of expression driven by 35S regulation. This is consistent with their apparent absence in transgenic plants expressing tagged AKIN10 under its endogenous promoter (Bitrián *et al.*, 2011). Alternatively, such intracellular localization may reflect a natural process only detectable under high expression levels. If this is the case, one possibility is that AKIN10 is normally delivered by the Golgi to the plasma membrane to be associated with β-subunits of SnRK1 that are *N*-myristolated specifically at this location (Pierre *et al.*, 2007).

### Does PTL alter the functioning of AKIN10 by influencing its intracellular localization?

We found that PTL bound AKIN10 and largely sequestered it to the nucleus in transient expression studies. Similar results have been reported for the FUSCA3 transcription factor (Tsai and Gazzarrini, 2012) and for the DUF581 family of nuclear proteins (Nietzsche *et al.*, 2014). A potential mechanism is that PTL and FUS3 at least, by binding to the C-terminal half of AKIN10, mask the conserved NES in this region, thus helping retain it in the nucleus.

In yeast, it is significant that activation of the cytoplasmic α-subunit Snf1 by glucose limitation results in its move to the nucleus in association with the β-subunit Gal83 (Hedbacker and Carlson, 2006), where they may regulate expression of many target genes.

However, it is not yet known if nuclear relocalization of AKIN10 has a similar functional significance. AKIN10 clearly has cytoplasmic functions. First, it seems likely that α-kinase subunits of SnRK1 (including AKIN10) aggregate with β- and βγ-subunits (López-Paz *et al.*, 2009; Ramon *et al.*, 2013) in the cytoplasm, and are activated there by phosphorylation. Secondly, direct target enzymes are likely to be phosphorylated in the cytoplasm. However, despite these cytoplasmic processes, a role for AKIN10 in the nucleus is implied by its nuclear localization seen in some circumstances (discussed above), and by its role in the large-scale reorganization of transcriptional networks (Baena-González *et al.*, 2007).

In conclusion, PTL can undergo a specific interaction with AKIN10 in the nucleus, but further studies are required to establish if this is part of a joint signalling process that dampens growth in the inter-sepal zone where energy is potentially limiting.

## Supplementary data

Supplementary data are available at *JXB* online.

Supplementary Table S1. Primers used in the study.

Supplementary Fig. S1. GUS reporter gene expression in flowers and inflorescences driven by AKIN10 regulatory sequences.

Supplementary Movie S1. Time-lapse series of a confocal optical section through a leaf of *Nicotiana benthamiana* transiently expressing 35S:YFP–AKIN10 and 35S:CFP–G-ck Golgi markers. Images were collected at 30 s intervals over 5min.

Supplementary Data

## Abbreviations:

3-AT: 3-amino-1,2,4-triazole
AD: activation domain
BD: binding domain
CFP: cyan fluorescent protein
GUS: β-glucuronidase
MILo: modification of intracellular localization
NES: nuclear export sequence
RT-PCR: reverse transcription-PCR
YFP: yellow fluorescent protein.
