# Supplementary material for: PETAL LOSS, a trihelix transcription factor that represses growth in Arabidopsis thaliana, binds the energy-sensing SnRK1 kinase AKIN10
Source: J Exp Bot. 2015 Feb 19;66(9):2475–85. doi: 10.1093/jxb/erv032 (PMC4986862; doi:10.1093/jxb/erv032)
Supplement: Supplementary Data [file supp_66_9_2475__index.html]

PETAL LOSS, a trihelix transcription factor that represses growth in Arabidopsis thaliana, binds the energy-sensing SnRK1 kinase AKIN10 — PETAL LOSS, a trihelix transcription factor that represses growth in Arabidopsis thaliana, binds the energy-sensing SnRK1 kinase AKIN10 — Supplementary Data 

# PETAL LOSS, a trihelix transcription factor that represses growth in *Arabidopsis thaliana*, binds the energy-sensing SnRK1 kinase AKIN10

## Supplementary Data

Data files

**Files in this Data Supplement:**

- Supplementary Data - Supplementary Data
- Supplementary Data - Supplementary Data
