# Supplementary material for: PETAL LOSS, a trihelix transcription factor that represses growth in Arabidopsis thaliana, binds the energy-sensing SnRK1 kinase AKIN10
Source: J Exp Bot. 2015 Feb 19;66(9):2475–85. doi: 10.1093/jxb/erv032 (PMC4986862; doi:10.1093/jxb/erv032)
Supplement: Supplementary Data [file supp_erv032_jexbot137703_file002.pdf]

Supplementary Data

PETAL LOSS, a trihelix transcription factor that represses growth in *Arabidopsis thaliana*, binds the energy-sensing SnRK1 kinase AKIN10

Martin O'Brien, Ruth N. Kaplan-Levy, Tezz Quon, Pia G. Sappl and David R Smyth*
School of Biological Sciences, Monash University, Melbourne, Vic. 3800, Australia

*For correspondence (e-mail david.smyth@monash.edu)

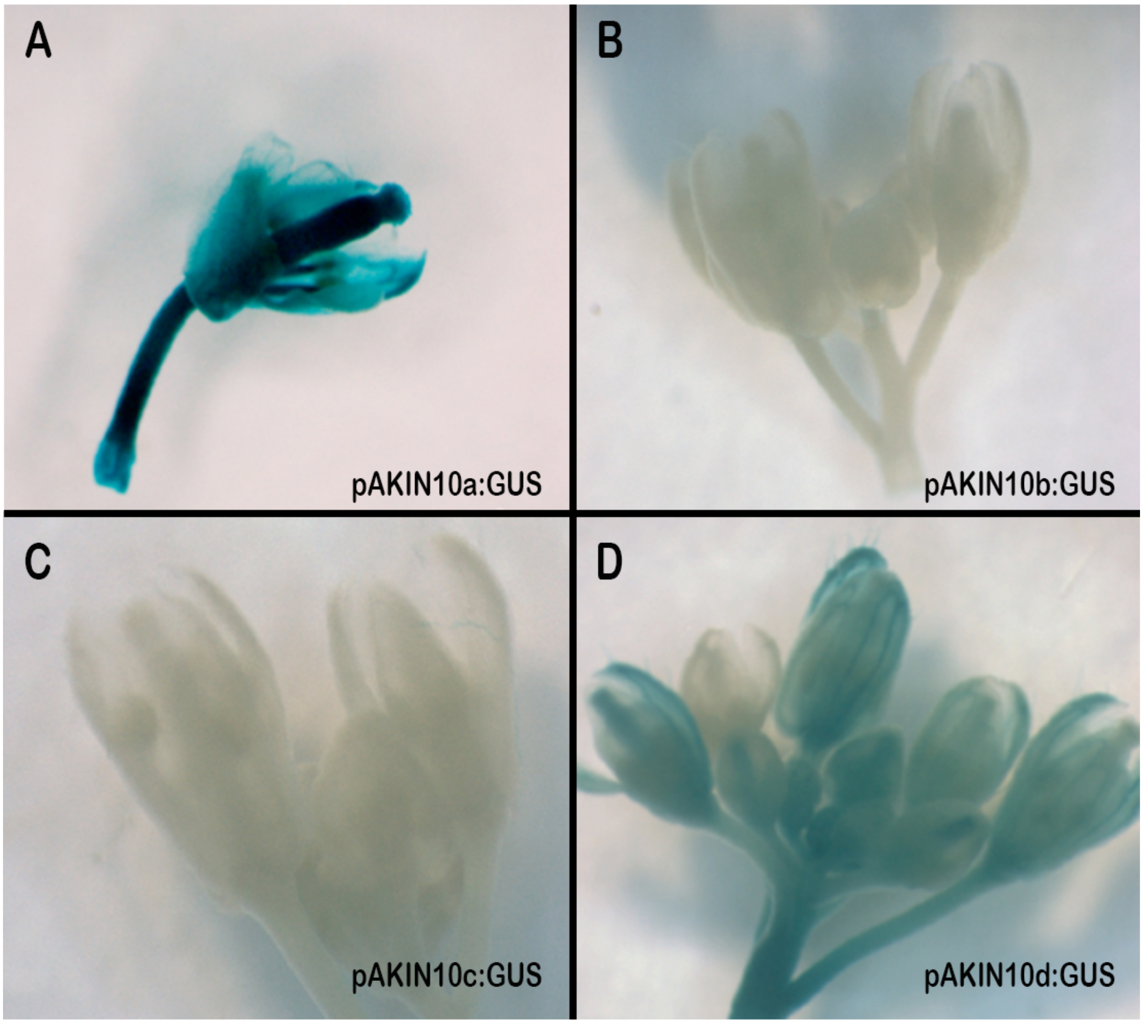

Fig. S1. GUS reporter gene expression in flowers and inflorescences driven by AKIN10 regulatory sequences

A representative T1 plant from 12 independent transformants is shown for each construct. GUS sequences were fused in frame to the first methionine of the second exon of AKIN10 in each case (see Fig. 3A).

(A) Strong expression occurs when driven by the full construct containing the 1,035 bp region extending from the stop codon of the upstream gene.

(B) No expression is detected when expression is driven by the 5' half of the full construct (extending 526 bp to the transcription start site of AKIN10).

(C) No expression is detected when expression is driven by the 232 bp of this extending from the last base of the 3' UTR of the upstream gene to the transcription start site of AKIN10.

(D) Weak expression occurs when driven by the 3' half of the full construct (the 509 bp region from the transcription start site, including the 5' UTR, first exon and intron of AKIN10).

Table S1. *Primers used in the study.*

Underlines shows artificial restriction sites added at the end of the amplified *Arabidopsis* sequences

A. Isolation of AKIN10 coding sequence from plasmid U24028

Primer name	Sequence
AKIN10_1f	5'-CTCTCTTTTTTTGTAGAGAATGG-3'
AKIN10_2r	5'-ATATAACTTGCCCGAAATTACC-3'

B. Isolation of genomic clone of AKIN10
(including upstream promoter, 5' UTR, first exon and first intron)

Primer name	Sequence
AKIN10_P1f	5'-GAGAGAATTCGATCACCTTTTTACTTGAGC-3'
AKIN10_P2r	5' GAGAAAGCTTCCCACTTCTACTGCCTGTGC 3'

C. Insertion of His-AKIN10 into *E. coli* expression vector pQE30

Primer name	Sequence
AKIN10_pQE30f	5'-GAGAGGATCCATGGATGGATCAGGCACAGG-3'
AKIN10_pQE30r	5'-GAGACTGCAGTCAGAGGACTCGGAGCTGAG-3'
